# A Multifunctional N-Doped Cu–MOFs (N–Cu–MOF) Nanomaterial-Driven Electrochemical Aptasensor for Sensitive Detection of Deoxynivalenol

**DOI:** 10.3390/molecules26082243

**Published:** 2021-04-13

**Authors:** Xiaoyan Wen, Qingwen Huang, Dongxia Nie, Xiuying Zhao, Haojie Cao, Wenhui Wu, Zheng Han

**Affiliations:** 1 College of Food Science & Technology, Shanghai Ocean University, Shanghai 201306, China; m180300715@st.shou.edu.cn (X.W.); m190310871@st.shou.edu.cn (X.Z.); m190310859@st.shou.edu.cn (H.C.); hanzheng@saas.sh.cn (Z.H.); 2 Shanghai Key Laboratory of Protected Horticultural Technology, Laboratory of Quality and Safety Risk Assessment for Agro-Products (Shanghai), Institute for Agro-Food Standards and Testing Technology, Ministry of Agriculture, Shanghai Academy of Agricultural Sciences, Shanghai 201403, China; huangqingwen@zju.edu.cn

**Keywords:** N-doped Cu–MOFs (N–Cu–MOF), deoxynivalenol, electrochemical aptasensor, wheat

## Abstract

Deoxynivalenol (DON) is one of the most common mycotoxins in grains, causing gastrointestinal inflammation, neurotoxicity, hepatotoxicity and embryotoxicity, even at a low quantity. In this study, a facile electrochemical aptasensor was established for the rapid and sensitive determination of DON based on a multifunctional N-doped Cu-metallic organic framework (N–Cu–MOF) nanomaterial. The N–Cu–MOF, with a large specific surface area and good electrical conductivity, served not only as an optimal electrical signal probe but also as an effective supporting substrate for stabilizing aptamers through the interactions of amino (-NH_2_) and copper. Under the optimal conditions, the proposed sensor provided a wide linear concentration range of 0.02–20 ng mL^−1^ (R^2^ = 0.994), showing high sensitivity, with a lower detection limit of 0.008 ng mL^−1^, and good selectivity. The sensor’s effectiveness was also verified in real spiked wheat samples with satisfactory recoveries of 95.6–105.9%. The current work provides a flexible approach for the rapid and sensitive analysis of highly toxic DON in food samples and may also be easily extended to detect other hazardous substances with alternative target-recognition aptamers.

## 1. Introduction

Deoxynivalenol (DON), a toxic secondary metabolite produced by the *Fusarium* genus, is one of the most frequently found mycotoxins in various foodstuffs, particularly wheat. DON is also called the vomiting toxin and results in acute and chronic toxicities in humans and animals, even at low concentrations. Symptoms include diarrhea, vomiting, gastrointestinal inflammation, anorexia, neurotoxicity and embryotoxicity [1,2,3]. To protect human health, the European Union (EU) has established a maximum DON limit of 1750 µg kg^−1^ both in wheat and maize, and the US Food and Drug Administration (FDA) has suggested an action level of 1000 µg kg^−1^ for DON in foods intended for human consumption [4,5]. Therefore, it is critical to develop a sensitive and rapid detection method to monitor DON in food.

A wide range of chromatographic approaches has been established for the detection of DON, including gas chromatography (GC) [6], high-performance liquid chromatography (HPLC) [7] and liquid chromatography-mass spectrometry (LC–MS) [8,9]. These methods are highly sensitive, accurate, and reliable. However, most are time-consuming and require complex sample preprocessing procedures, professional instrument operators, and expensive equipment. To solve these problems, rapid detection techniques for DON are emerging, such as enzyme-linked immunosorbent assay (ELISA) [10], lateral flow immunoassay (LFI) [11], dual near-infrared fluorescence-based lateral flow immunosensor [12], immunochromatographic test strip [13], multiplex luminescent detection [14] and electrochemical biosensors. Among these, electrochemical biosensors have received increasing attention due to the advantages of simple operation, high sensitivity, low cost and portability. As an effective recognition element in electrochemical biosensors, aptamers are widely used and have the advantages of specific recognition of target, simple synthesis, cost-effectiveness, good stability, and convenient storage [15].

The concentrations of DON are frequently low in crops, and therefore, subsurface materials that can enlarge the surface area, improve the conductivity and steadily immobilize a large number of aptamers are essential for constructing sensitive and selective immunosensors. A metallic organic framework (MOF) is a crystalline material composed of inorganic metal centers (metal ions, clusters) assembled with organic ligands. It has a large surface area, high porosity, excellent catalytic performance, rich active sites, and an unrivaled degree of tunability [16]. A variety of MOF materials have been applied to construct electrochemical sensors, including copper-based MOF (Cu–MOF) [17], zirconium-based MOF [18], nickel-based MOF [19] and cobalt-based MOF [20]. Among these, Cu–MOF, due to its superior electrocatalytic features [21] and excellent electrical signals generated from the variable valence of Cu [22], has been extensively applied in electrochemical sensors for the detection of glucose, hepatitis B virus DNA, patulin, etc. [23,24,25]. However, these Cu–MOF crystals frequently show irregular shapes, large particle sizes and poor conductivity. To further endow additional functionalities and improve its performance, the modification of Cu–MOF (by the introduction of heteroatoms, functional groups, and metal ions) is an effective strategy [26]. Cu–MOF doped with nitrogen (N–Cu–MOF), as a novel material, has attracted increasing attention due to its controllable architectural excellence, well-defined order and high electrochemical activity [27]. However, sensors using N–Cu–MOF have rarely been reported and remain at an early stage.

**Figure 1:**
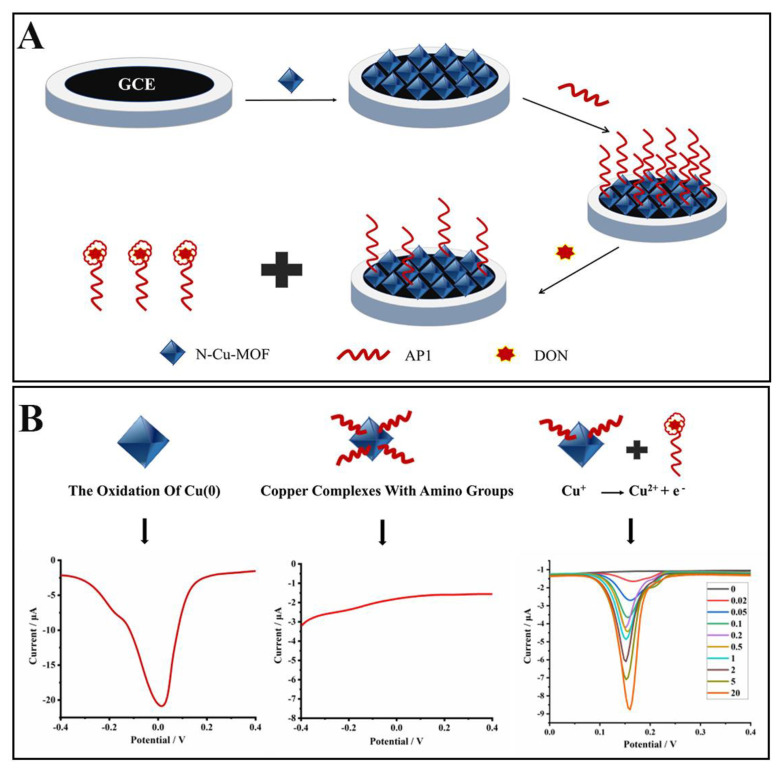
Schematic illustration of the electrochemical aptasensor based on the N–doped Cu–metallic organic framework (N–Cu–MOF) for selective detection of deoxynivalenol (DON). (**A**) Preparation of the aptasensor. (**B**) Mechanisms of the sensing system.

In the current research, a novel N–Cu–MOF-based aptasensor was successfully developed for the quick, highly sensitive and selective detection of DON (Figure 1). For the first time, N–Cu–MOF was not only used as an effective supporting material, which facilitated accommodation with a large number of aptamers, thus greatly improving the stability and the selectivity, but also served as an electrochemical probe with excellent electrical signals, significantly enhancing the sensitivity of the established sensor. The current work is expected to provide a promising and novel strategy for the construction of aptasensors with good performance for DON detection, which also could be easily extended to other hazardous compounds.

## 2. Results

### 2.1. Characterization of the N–Cu–MOF

**Figure 2:**
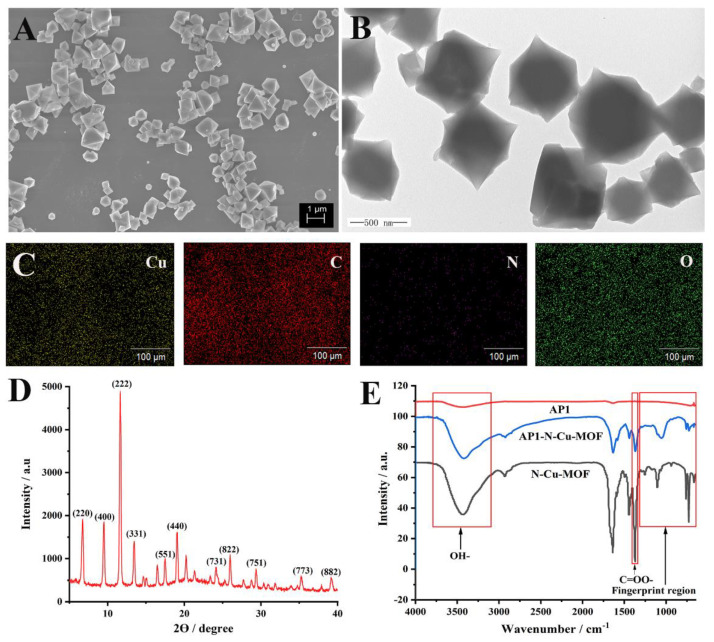
(**A**) Scanning electron microscopy image (SEM), (**B**) transmission electron microscopy image (TEM), (**C**) elemental mapping and (**D**) X-ray diffraction patterns (XRD) of N–Cu–MOF; (**E**) Fourier-transform infrared spectroscopy (FT-IR) spectra of N–Cu–MOF, aptamer (AP1) and AP1–N–Cu–MOF.

The morphology and microstructures of the N–Cu–MOF were characterized by scanning electron microscope (SEM) and transmission electron microscope (TEM). The prepared N–Cu–MOF nanoparticles with regular octahedral crystal structures were uniformly distributed (Figure 2A,B). In AP1/N–Cu–MOF (Figure S1), DNA molecules are clearly located on the scaffolds of N–Cu–MOF, resulting in vague crystal edges. The average diameter of the N–Cu–MOF crystallite was roughly 540 nm. In the element mapping image of N–Cu–MOF (Figure 2C), Cu, C, N and O elements were clearly observed, indicating the successful incorporation of nitrogen elements into the synthesized nanoparticles. In addition, the X-ray powder diffraction (XRD) pattern of N–Cu–MOF (Figure 2D) presented the characteristic peaks at 2θ = 11.6°, 13.4°, 19.0° and 23.3°, which were consistent with the previously reported HKUST-1-type Cu-based MOF crystals, with the exception of some changes of Bragg intensities [27]. The high diffraction intensities and sharp peaks in XRD patterns of N–Cu–MOF (Figure 2D) confirmed the high crystallinity of the synthesized nanomaterials, indicating that the framework structure of Cu–MOF was not destroyed by the introduction of poly(vinylpyrrolidone) (PVP) in the preparation stage [28].

Furthermore, the Fourier-transform infrared (FT-IR) spectra of PVP, ligand 1,3,5-benzenetricarboxylic acid (H_3_BTC) and N–Cu–MOF were investigated, as shown in Figure S2. The effective coordination of the carboxylate moiety of the H_3_BTC ligand with metal ions was confirmed by the asymmetric (1639 and 1446 cm^−1^) and symmetric (1368 cm^−1^) vibrations of the C=OO- group in N–Cu–MOF. In addition, compared with PVP, it was observed that N–Cu–MOF had analog characteristic bands at 2750–3080 cm^−1^ (C-H stretching). The bands in the regions of 3100–3800 cm^−1^ and 729–761 cm^−1^ were assigned to the OH-bond stretching of the surface’s active carbon and the phenyl C-H bending, respectively. All of these results matched well with those of reported HKUST-1 crystals [27,29], demonstrating the successful preparation of N–Cu–MOF.

FT-IR was also employed to confirm the interactions between AP1 and N–Cu–MOF (Figure 2E). The fingerprint region (1300–650 cm^−1^) in the infrared spectrum of AP1–N–Cu–MOF was significantly different from that of N–Cu–MOF and AP1, proving the successful complexation of the aptamer and N–Cu–MOF [30]. The FT-IR absorption peaks at around 3100–3800 cm^−1^ (OH-), 729–761 cm^−1^ (C–H) and 1368 cm^−1^ (C=OO-) were observed in AP1–N–Cu–MOF and N–Cu–MOF, verifying the good maintenance of the structure of N–Cu–MOF.

The influence of the amino groups and copper for the stabilization of the aptamers was further explained by investigating the UV-vis spectra of N–Cu–MOF, AP1 and AP1–N–Cu–MOF. A characteristic UV-vis absorption peak at 258 nm was clearly observed for AP1, whereas N–Cu–MOF showed no obvious UV-vis adsorptions (Figure S3). Regarding AP1–N–Cu–MOF, the typical peak of 258 nm was blue-shifted to 230 nm, verifying the complexion of the amino groups in AP1 with the copper in N–Cu–MOF for the stabilization of the aptamers.

X-ray photoelectron spectroscopy (XPS) measurements were also performed for AP1–N–Cu–MOF. Two peaks appeared at 933.6 and 953.4 eV, which corresponded to the 2p_3/2_ and 2p_1/2_ peaks of Cu^+^ and Cu (0), respectively (Figure S4).

Nitrogen adsorption–desorption analysis (Figure S5) was also carried out to determine the Brunauer–Emmett–Teller (BET)-specific surface area and pore size of N–Cu–MOF. The results showed that the BET-specific surface area and average pore size of N–Cu–MOF were 1855 m^2^ g^−1^ and 2.42 nm, respectively, verifying the large specific surface area of the synthesized nanomaterials, which could serve as an effective electrochemical sensing material.

### 2.2. Electrochemical Behaviors of Fabricated Electrodes

**Figure 3:**
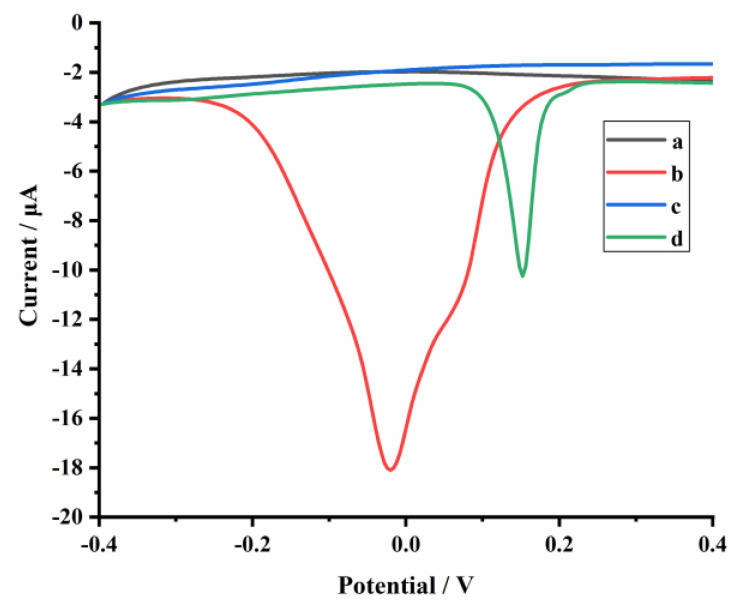
Differential pulse voltammetry (DPV) measurements of the glassy carbon electrode (GCE) with different modifications: (**a**) GCE, (**b**) N–Cu–MOF/GCE, (**c**) AP1/N–Cu–MOF/GCE in 1 mmol L^−1^ PBS (pH 5.0) and (**d**) AP1/N–Cu–MOF/GCE is in PBS (1 mmol L^−1^, pH 5.0) containing 10 ng mL^−1^ DON.

The electrochemical behaviors of the aptasensor were investigated in PBS (1 mmol L^−1^, pH 5.0). As shown in Figure 3, compared to a bare glassy carbon electrode (GCE) (curve a), an obvious peak appeared at around −0.02 V when the GCE was modified with N–Cu–MOF nanoparticles (curve b), which was ascribed to the electrochemical oxidation of Cu (0) [31,32]. The electrical signal of N–Cu–MOF disappeared when the electrode was further assembled with aptamer (AP1) due to the complexation of amino groups (-NH_2_) in AP1 with copper. After incubation of the AP1-grafted N–Cu–MOF/GCE into DON solution (10 ng mL^–1^), a new oxidation peak at about 0.15 V appeared (curve d), implying the restoration of electrochemical activity of copper-based nanomaterial after the removal of AP1 from the N–Cu–MOF-modified electrode. The peak from 0.15 V belonged to the oxidation of Cu^+^: Cu^+^ →Cu^2+^ + e^−^, which is consistent with the previous publications [33]. The anodic peak may be related to the change of the valence state of Cu. The peak current at 0.15 V increased as the amount of the aptamer located on the electrode decreased, which was attributed to the nonconductive aptamer increasingly blocking the electron transfer by N–Cu–MOF. When detecting DON, aptamers were removed from N–Cu–MOF; thus, the signal appeared and was proportional to the concentration of DON.

### 2.3. Optimization of Assay Conditions

Several key parameters, including the amount of N–Cu–MOF and AP1, the incubation time of the aptasensor in the PBS buffer containing DON, and the PBS buffer’s pH value, were all thoroughly investigated to achieve optimal electrochemical performance.

The loading amount of N–Cu–MOF as supporting material and the electrical signal probe are critical to improving the electrochemical performance. Therefore, 5 µL of various concentrations of N–Cu–MOF solutions (2, 3, 4 and 5 mg mL^−1^) were investigated to prepare the AP1/N–Cu–MOF/GCE electrode. The highest differential pulse voltammetry (DPV) response was obtained with 4 mg mL^−1^ (Figure S6A). Then, 5 µL of different concentrations of AP1 solutions (4, 6, 8, 10 and 12 μmol L^−1^) were also evaluated in 2 ng mL^−1^ DON solution. As shown in Figure S6B, the DPV response reached the maximum value when the concentration of AP1 was 10 μmol L^−1^. The pH value of electrolyte solution was also investigated, and the DPV measurement showed that the peak currents increased with the pH values from 4.0 to 5.0, then decreased from 5.0 to 8.0 (Figure S6C). The highest peak current was achieved at pH 5.0, which could be attributed to the proton-coupled electron transfer occurring in N–Cu–MOF [34]. As a result, pH 5.0 PBS was chosen as the optimal electrolyte solution for DON detection. When the established sensor was used for 2 ng mL^−1^ DON detection, the DPV response magnified with the incubation time from 10 to 40 min and then reached a plateau (Figure S6D). Hence, 40 min was chosen as the optimal incubation time.

### 2.4. Analytical Performance of the Optimized Electrochemical Aptasensor

**Figure 4:**
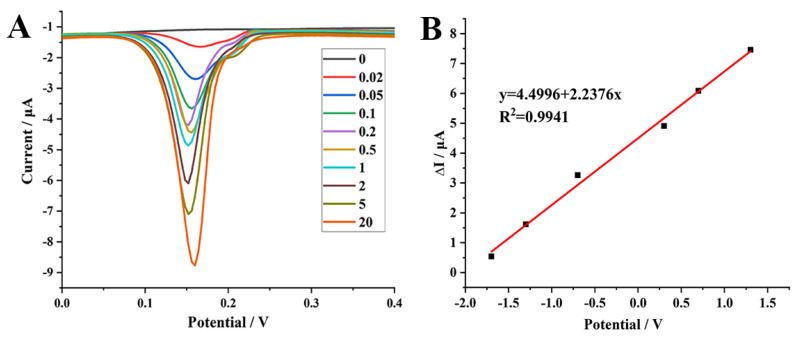
(**A**) differential pulse voltammetry (DPV) curves recorded on AP1/N–Cu–MOF/GCE with the successive addition of DON in PBS (1 mmol L^−1^, pH 5.0). (**B**) Calibration curves of DON with AP1/N–Cu–MOF/GCE as the working electrode. ΔI = I_p_−I_p0_, where I_p0_ is the peak current for the blank PBS and I_p_ represents the peak current for PBS containing DON.

Under the optimal experimental conditions, the current responses increased gradually with the increase in DON level (Figure 4A). Furthermore, a good linear relationship was obtained between the change of the oxidation peak currents (ΔI) and the logarithm of DON concentrations in the range of 0.02–20 ng mL^−1^ (Figure 4B). The linear regression equation was ΔI (μA) = 4.4996 + 2.2376 lgC_DON_ (ng mL^−1^), with a correlation coefficient (R^2^) of 0.9941, and the detection limit was 0.008 ng mL^−1^ (S/N = 3).

### 2.5. Selectivity and Reproducibility

**Figure 5:**
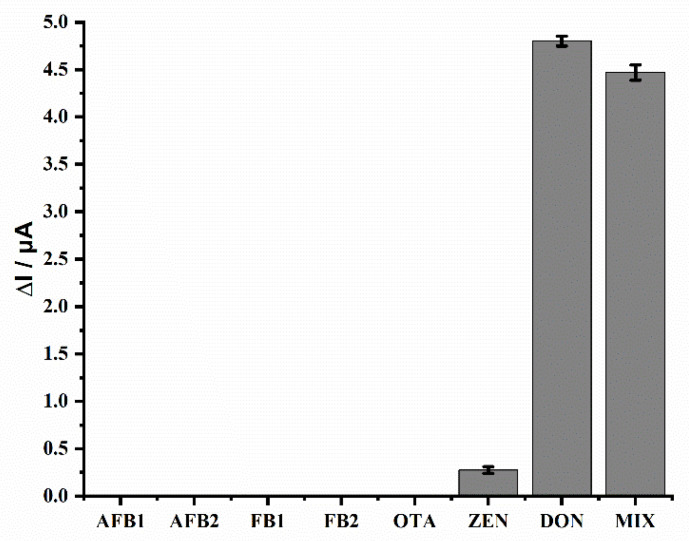
Selectivity of the electrochemical aptasensor in the detection of DON. The concentration was 2 ng mL^−1^ for all mycotoxins. MIX represents a mixture of multiple mycotoxins: AFB1+ AFB2+ FB1+ FB2+ OTA+ ZEN+ DON.

The selectivity of the electrochemical aptasensor was evaluated by the determination of DON and other frequently co-occurring mycotoxins, including aflatoxin B1 (AFB1), aflatoxin B2 (AFB2), fumonisin B1 (FB1), fumonisin B2 (FB2), zearalenone (ZEN) and ochratoxin A (OTA) [35]. The concentration of all analytes was 2 ng mL^−1^. As shown in Figure 5, negligible signal responses were observed when different interferences were analyzed, indicating that these commonly co-existing mycotoxins have no significant effects on the N–Cu–MOF-based aptasensor. Considering that a sample may contain multiple mycotoxins, we also investigated the recognition performance of DON in the presence of various interfering substances. Even when different mycotoxins were mixed together, the influence of these interfering substances on the detection of DON was insignificant. These results indicate that the designed aptasensor possesses satisfactory selectivity for the detection of DON due to the high specific binding of AP1 to DON.

To prove the reliability of the established sensor, its reproducibility was also studied. The signal changes of the DPV measurements using five different batches of sensors modified with N–Cu–MOF were in the range of 15.56 μA–16.41 μA (Figure S7), with a relative standard deviation (RSD) of 2.1%. In addition, the DPV responses of five different AP1/N–Cu–MOF-modified electrodes to 2 ng mL^−1^ DON were studied, and the RSD was 6.43%. All of these results confirmed the acceptable reproducibility of the established electrode. To further characterize the advantages, the proposed sensor was compared to the previously reported methods. Excellent analytical performance with much higher detection limit (LOD) values was obtained, as shown in Table 1. However, this aptasensor was not suitable for recycling, and reversible sensors will be investigated in our future research.

### 2.6. Applications to Real Samples

To further evaluate its feasibility, the AP1/N–Cu–MOF-based electrochemical sensor was applied to detect DON in real wheat samples. Blank wheat samples (DON-free) were spiked with different concentrations of DON (0.05, 0.5, 2.5, 5 μg kg^−1^) in triplicate (n = 3), and three additional blank samples (DON-free) were tested as the control. As shown in Table 2, satisfactory recoveries in the range of 95.6–105.9% were obtained with RSDs of 3.54–6.01%, demonstrating the sensor’s feasibility and exhibiting a considerable application potential to detect DON in a complex sample matrix.

## 3. Materials and Methods

### 3.1. Materials and Instruments

H_3_BTC was purchased from Shanghai Titan Technology Co. Ltd. (Adamas, Shanghai, China). PVP (average M.W. K17) was purchased from Beijing Bailingwei Technology Co., Ltd. (damas-beta, Beijing, China). Copper (II) nitrate (Cu (NO_3_)_2_·3H_2_O), N, N-dimethylformamide (DMF, ≥99.8%) and sulfuric acid (H_2_SO_4_) were all obtained from Sigma-Aldrich Co. Ltd. (St Louis, MO, USA).

DON, AFB1, AFB2, FB1, FB2, ZEN and OTA were purchased from Sigma-Aldrich (St. Louis, MO, USA). Wheat samples were collected from Shanghai Pujiang Storage Co. Ltd. (Shanghai, China). The oligonucleotide sequence of the aptamers specific for DON was selected according to the previous reports [39]: 5′–C6–GCATCACTACAGTCATTACGCATCGTAGGGGGGATCGTTAAGGAAGTGCCCGGAGGCGGTATCGTGTGAAGTGCTGTCCC–3′ (AP1, specific for DON), which was synthesized by Sangon Biotech Co. Ltd. (Shanghai, China).

The synthesized nanomaterials were characterized by a ZEISS Sigma 500/VP SEM (Carl Zeiss AG Co., Jena, Germany) and a JEM-1200EX TEM (JEOL Ltd., Tokyo, Japan). XRD spectra were collected on a Bruker D8 Advance XRD (Bruker Technology Co., Ltd., Karlsruhe, Germany). The composition of the synthesized nanomaterials was analyzed by energy dispersive spectroscopy (EDS, Thermo Fisher Scientific Inc., Bartlesville, OK, USA). Nicolet iS10 FT-IR (Thermo Fisher Scientific Inc.) was used to determine the functional group in the chemical structure of the synthesized materials at the frequency range of 500–4000 cm^−1^. Nitrogen adsorption–desorption analysis was performed using an automated gas sorption analyzer (Autosorb-IQ, Konta Instruments Inc., Florida, FL, USA). UV-vis absorption spectroscopy was performed on a NanoDrop 2000/2000c (Genomics Ltd., Shenzhen, China). XPS was performed on an AXIS Supra (Kratos Analytical Ltd., Stretford, UK).

All the electrochemical measurements were acquired with a CHI660D electrochemical workstation (Chenhua Instruments Co., Shanghai, China). A conventional three-electrode system was used. A glassy carbon electrode (GCE, 3.0 mm diameter) was used as the working electrode, and a saturated calomel electrode and a Pt electrode were used as the reference electrode and the counter electrode, respectively.

### 3.2. Synthesis of N-Doped Cu–MOF (N–Cu–MOF)

The N–Cu–MOF was prepared, referring to the related literature [34]. Briefly, 0.875 g Cu (NO_3_)_2_·3H_2_O and 0.3 g PVP were dissolved in DMF solution and stirred for 5 min to obtain a homogenous solution. Then, 0.42 g H_3_BTC was added to the solution, which was stirred for 10 min. Subsequently, the mixture was transferred into a 100 mL Teflon-lined stainless-steel autoclave, which was placed in a furnace at 80 °C for 24 h. The obtained blue solid was collected by centrifugation, washed with fresh DMF twice and ethanol three times, and then dried at 60 °C for 12 h.

### 3.3. Fabrication of the Electrochemical Aptasensor

The GCE was successively polished with 1, 0.3 and 0.05 μm alumina powder and then washed with ultrapure water. N–Cu–MOF powders were dispersed in deionized water and ultrasonicated for 30 min to obtain a homogeneous dispersion (4 mg mL^−1^). Afterward, an aliquot (5 μL) of the N–Cu–MOF aqueous dispersion was coated onto the electrode surface and dried at room temperature. The electrode was then gently rinsed with PBS (1 mmol L^−1^, pH 5.0) to remove poorly adhered N–Cu–MOF and dried. After this, 5 μL of AP1 (10 μmol L^−1^) was dropped on the modified electrode surface and incubated for 12 h at 37 °C to conjugate AP1 with N–Cu–MOF. The obtained AP1-grafted N–Cu–MOF/GCE was also rinsed with PBS (1 mmol L^−1^, pH 5.0) to remove any unbound AP1 and stored at 4 °C until use.

### 3.4. Electrochemical Measurements

For the detection of DON, differential pulse voltammetry (DPV) measurements were performed in 5 mL f PBS (1 mmol L^−1^, pH 5.0) with the experimental parameters as follows: voltage scan range was from −0.4 V to 0.4 V, and the amplitude, pulse width and pulse period were 0.05 V, 0.05 s and 0.5 s, respectively. The current difference (ÄI) was calculated according to Equation (1):

ΔI = I_p_–I_p0_ (1)

where I_p__0_ is the peak current for the blank PBS and I_p_ represents the peak current for PBS containing DON.

### 3.5. Sample Preparation

The wheat flour samples (2.0 g) were macerated with 10 mL of acetonitrile/water (84:16, *v/v*) for 5 min, followed by ultrasonic extraction for 40 min. After centrifugation at 8000 r/min for 5 min, an aliquot of 100 μL of supernatant was collected, diluted with 9.9 mL of PBS (1 mmol L^−1^, pH 5.0), and then analyzed with the proposed aptasensor.

## 4. Conclusions

In this study, we successfully prepared an electrochemical aptasensor based on N–Cu–MOF for the rapid detection of DON for the first time. The multifunctional N–Cu–MOF nanomaterials with a large specific surface area were not only used as an effective supporting platform but also as a sensitive electrical signal probe. The present study determined the signals generated from the variable valence of Cu and revealed the signal changes under different experimental conditions. Under optimized experimental conditions, the proposed sensor showed a wide linear range, low detection limit, high selectivity and good reproducibility and was successfully applied for the rapid detection of DON in real wheat samples. In addition, the proposed strategy could be easily expanded to other mycotoxins or hazardous materials by altering specific aptamers.

## Figures and Tables

**Table 1:** Comparison of DON determination using different electrochemical sensors.

Materials	Signal Molecule	Bioreceptors	LOD	Linear Range	Ref
Gold nanoparticles and polypyrrole -electrochemically reduced graphene oxide nanocomposite film	[Fe (CN)_6_]^3–/4–^	Antibody	8.6 ng mL^−1^	100–4500 ng mL^−1^	[36]
Molecular imprinting	QD@SiO_2_	Molecular imprinting	35 ng mL^−1^	55–420 ng mL^−1^	[14]
Screen-printed gold electrode	[Fe (CN)_6_]^3–/4–^	Molecular imprinting	0.3 ng mL^−1^	5–500 ng mL^−1^	[37]
Polyaniline and gold nanoparticles	[Fe (CN)_6_]^3–/4–^	Aptamer	3.2 ng mL^−1^	0–50 ng mL^−1^	[38]
Iron nanoflorets graphene nickel	--	Aptamer	2.11 pg mL^−1^	1 fg mL^−1^– 1 ng mL^−1^	[39]
N–Cu–MOF	N–Cu–MOF	Aptamer	0.008 ng mL^−1^	0.02–20 ng mL^−1^	This work

**Table 2:** DON recoveries in wheat samples analyzed by the proposed aptasensor platform (n = 3).

Analytes	Added (μg kg^−1^)	Found (μg kg^1^)	Recovery (%)	RSD (%)
DON	0.05	0.051	101.5	5.59
	0.50	0.530	105.9	3.54
	2.50	2.389	95.6	4.70
	5.00	4.990	99.8	6.01

## Supplementary Materials

The following are available online. Figure S1: Transmission electron microscopy (TEM) of AP1–N–Cu–MOF; Figure S2: Fourier-transform infrared (FT-IR) spectra of N–Cu–MOF, 1,3,5-benzenetricarboxylic acid (H_3_BTC) and polyvinylpyrrolidone (PVP); Figure S3: The UV-vis spectrum of the N–Cu–MOF and AP1–N–Cu–MOF; Figure S4: X-ray photoelectron spectroscopy (XPS) of AP1–N–Cu–MOF; Figure S5: N_2_ adsorption/desorption isotherms of N–Cu–MOF; Figure S6: Optimization of AP1–N–Cu–MOF/GCE-based aptasensor. DPV responses with different concentrations of N–Cu–MOF (A) and AP1 (B), different pH values (C) and different incubation times (D) of AP1–N–Cu–MOF/GCE electrode in 1 mmol L^−1^ PBS containing 2 ng mL^−1^ DON.

## Sample Availability

Not available.

## References

[1] Girolamo A.D., Ciasca B., Pascale M., Lattanzio V.M.T. (2020). Determination of zearalenone and trichothecenes, including deoxynivalenol and its acetylated derivatives, nivalenol, T-2 and HT-2 toxins, in wheat and wheat products by LC-MS/MS: A collaborative study. Toxins.

[2] Gerez J.R., Desto S.S., Bracarense A.P.F.R.L. (2017). Deoxynivalenol induces toxic effects in the ovaries of pigs: An ex vivo approach. Theriogenology.

[3] Wu Y., Yu J., Li F., Li J., Shen Z. (2020). A calibration curve implanted enzyme-linked immunosorbent assay for simultaneously quantitative determination of multiplex mycotoxins in cereal samples, soybean and peanut. Toxins.

[4] European Commission (2006). Commission recommendation No 2006/576/EC of 17 August 2006 on the presence of deoxynivalenol, zearalenone, ochratoxin A, T-2 and HT-2 and fumonisins in products intended for animal feeding. Off J. Eur. Union.

[5] U.S (2010). Food and Drug Administration (USFDA). Guidance for Industry and FDA: Advisory Levels for Deoxynivalenol (DON) in Finished Wheat Products for Human Consumption and Grains and Grain By-Products Used for Animal Feed..

[6] Olsson J., Borjesson T., Lundstedt T., Schnurer J. (2002). Detection and quantification of ochratoxin A and deoxynivalenol in barley grains by GC-MS and electronic nose. Int. J. Food Microbiol..

[7] Ok H.E., Lee S.Y., Chun H.S. (2018). Occurrence and simultaneous determination of nivalenol and deoxynivalenol in rice and bran by HPLC-UV detection and immunoaffinity cleanup. Food Control.

[8] Stastny K., Stepanova H., Hlavova K., Faldyna M. (2019). Identification and determination of deoxynivalenol (DON) and deepoxy-deoxynivalenol (DOM-1) in pig colostrum and serum using liquid chromatography in combination with high resolution mass spectrometry (LC-MS/MS (HR)). J. Chromatogr. B.

[9] Fernandez C., Stack M.E., Musser S.M. (1994). Determination of Deoxynivalenol in 1991 U.S. Winter and Spring Wheat by High-Performance Thin-Layer Chromatography. J. AOAC Int..

[10] Zhang Y., Yang J., Lu Y., Ma D.-Y., Qi M.G., Wang S. (2017). A competitive direct enzyme-linked immunosorbent assay for the rapid detection of deoxynivalenol: Development and application in agricultural products and feedstuff. Food Agric. Immunol..

[11] Urusov A.E., Gubaidullina M.K., Petrakova A.V., Zherdev A.V., Dzantiev B.B. (2017). A new kind of highly sensitive competitive lateral flow immunoassay displaying direct analyte-signal dependence. Application to the determination of the mycotoxin deoxynivalenol. Microchim. Acta.

[12] Jin Y., Chen Q., Luo S., He L., Fan R., Zhang S., Yang C., Chen Y. (2021). Dual near-infrared fluorescence-based lateral flow immunosensor for the detection of zearalenone and deoxynivalenol in maize. Food Chem..

[13] Huang X., Huang X., Xie J., Li X., Huang Z. (2020). Rapid simultaneous detection of fumonisin B1 and deoxynivalenol in grain by immunochromatographic test strip. Anal. Biochem..

[14] Beloglazova N., Lenain P., Tessier M., Goryacheva I., Hens Z., Saeger S.D. (2019). Bioimprinting for multiplex luminescent detection of deoxynivalenol and zearalenone. Talanta.

[15] Zhou W., Saran R., Liu J. (2017). Metal Sensing by DNA. Chem. Rev..

[16] Morozan A., Jaouen F. (2012). Metal organic frameworks for electrochemical applications. Energy Environ. Sci..

[17] Dang W.J., Sun Y.M., Jiao H., Xu L., Lin M. (2020). AuNPs-NH_2_/Cu-MOF modified glassy carbon electrode as enzyme-free electrochemical sensor detecting H_2_O_2_. J. Electroanal. Chem..

[18] He B., Dong X. (2021). Nb.BbvCI powered DNA walking machine-based Zr-MOFs-labeled electrochemical aptasensor using Pt@AuNRs/Fe-MOFs/PEI-rGO as electrode modification material for patulin detection. Chem. Eng. J..

[19] Gao F., Tu X., Ma X., Xie Y., Zou J., Huang X., Qu F., Yu Y., Lu L. (2020). NiO@Ni-MOF nanoarrays modified Ti mesh as ultrasensitive electrochemical sensing platform for luteolin detection. Talanta.

[20] He B., Yan X. (2020). Ultrasensitive electrochemical aptasensor based on CoSe_2_/AuNRs and 3D structured DNA-PtNi@Co-MOF networks for the detection of zearalenone. Sens. Actuators B: Chem..

[21] Murinzi T.W., Clement T.A., Chitsa V., Mehlana G. (2018). Copper oxide nanoparticles encapsulated in HKUST-1 metal-organic framework for electrocatalytic oxidation of citric acid. J. Solid State Chem..

[22] Cortés-Súarez J., Celis-Arias V., Beltrán H.I., Tejeda-Cruz A., Ibarra I.A., Romero-Ibarra J.E., Sánchez-González E., Loera-Serna S. (2019). Synthesis and Characterization of an SWCNT@HKUST-1 Composite: Enhancing the CO_2_ Adsorption Properties of HKUST-1. ACS Omega.

[23] Song Y., Xu M., Gong C., Shen Y., Wang L., Xie Y., Wang L. (2018). Ratiometric electrochemical glucose biosensor based on GOD/AuNPs/Cu-BTC MOFs/macroporous carbon integrated electrode. Sens. Actuators B: Chem..

[24] Lin X., Lian X., Luo B., Huang X.-C. (2020). A highly sensitive and stable electrochemical HBV DNA biosensor based on ErGO-supported Cu-MOF. Inorg. Chem. Commun..

[25] Hatamluyi B., Rezayi M., Beheshti H.R., Boroushaki M.T. (2020). Ultra-sensitive molecularly imprinted electrochemical sensor for patulin detection based on a novel assembling strategy using Au@Cu-MOF/N-GQDs. Sens. Actuators B: Chem..

[26] Kirchon A., Feng L., Drake H.F., Joseph E.A., Zhou H.-C. (2018). From fundamentals to applications: A toolbox for robust and multifunctional MOF materials. Chem. Soc. Rev..

[27] Chen S., Wang C., Zhang M., Zhang W., Qi J., Sun X., Wang L., Li J. (2020). N-doped Cu-MOFs for efficient electrochemical determination of dopamine and sulfanilamide. J. Hazard. Mater..

[28] Kumar R.S., Kulandainathan M.A. (2012). Highly selective electrochemical reduction of carbon dioxide using Cu based metal organic framework as an electrocatalyst. Electrochem. Commun..

[29] Xia H., Li Z., Zhong X., Li B., Jiang Y., Jiang Y. (2019). HKUST-1 catalyzed efficient in situ regeneration of NAD^+^ for dehydrogenase mediated oxidation. Chem. Eng. Sci..

[30] Aneja K.S., Bohm S., Khanna A.S., Bohm H.L.M. (2015). Graphene based anticorrosive coatings for Cr (VI) replacement. Nanoscale.

[31] Hosseini H., Ahmar H., Dehghani A., Bagheri A., Fakhari A.R., Amini M.M. (2013). Au-SH-SiO_2_ nanoparticles supported on metal-organic framework (Au-SH-SiO_2_@Cu-MOF) as a sensor for electrocatalytic oxidation and determination of hydrazine. Electrochim. Acta.

[32] Qiao X., Xia F., Tian D., Chen P., Liu J., Gu J., Zhou C. (2019). Ultrasensitive “signal-on” electrochemical aptasensor for assay of acetamiprid residues based on copper-centered metal-organic frameworks. Anal. Chim. Acta.

[33] Raj S.M., Reddy K. (2017). Cyclic voltammetric studies of the interaction of rizatriptan benzoate with copper in aqueous solution. Int. Res. J. Pharm..

[34] Singh S., Numan A., Zhan Y., Singh V., Hung T.V., Nam N.D. (2020). A novel highly efficient and ultrasensitive electrochemical detection of toxic mercury (II) ions in canned tuna fish and tap water based on a copper metalorganic framework. J. Hazard. Mater..

[35] Neme K., Mohammed A. (2017). Mycotoxin occurrence in grains and the role of postharvest management as a mitigation strategies A review. Food Control.

[36] Lu L., Seenivasan R., Wang Y.-C., Yu J.-H., Gunasekaran S. (2016). An electrochemical immunosensor for rapid and sensitive detection of mycotoxins fumonisin B1 and deoxynivalenol. Electrochim. Acta.

[37] Radia A.-E., Eissaa A., Wahdan T. (2019). Impedimetric sensor for deoxynivalenol based on electropolymerised molecularly imprinted polymer on the surface of screen-printed gold electrode. Int. J. Environ. Anal. Chem..

[38] Subak H., Selvolini G., Macchiagodena M., Ozkan-Ariksoysal D., Pagliai M., Procacci P., Marrazza G. (2020). Mycotoxins aptasensing: From molecular docking to electrochemical detection of deoxynivalenol. Bioelectrochemistry.

[39] Ong C.C., Sangu S.S., Illias N.M., Gopinath S.C.B., Saheed M.S.M. (2020). Iron nanoflorets on 3D-graphene-nickel: A ‘Dandelion’ nanostructure for selective deoxynivalenol detection. Biosens. Bioelectron..

