# Supplementary material for: A Multifunctional N-Doped Cu–MOFs (N–Cu–MOF) Nanomaterial-Driven Electrochemical Aptasensor for Sensitive Detection of Deoxynivalenol
Source: Molecules. 2021 Apr 13;26(8):2243. doi: 10.3390/molecules26082243 (PMC8069659; doi:10.3390/molecules26082243)
Supplement: Supplementary file 1 [file molecules-26-02243-s001.pdf]

Supporting Information

Article

A Multifunctional N-Doped Cu–MOFs (N–Cu–MOF) Nanomaterial-Driven Electrochemical Aptasensor for Sensitive Detection of Deoxynivalenol

Xiaoyan Wen [1,2,†], Qingwen Huang [2,†], Dongxia Nie [1,2,*], Xiuying Zhao [1,2], Haojie Cao [1,2], Wenhui Wu [1,*] and Zheng Han [1,2]

[1] College of Food Science & Technology, Shanghai Ocean University, Shanghai 201306, China; m180300715@st.shou.edu.cn (X.W.); m190310871@st.shou.edu.cn (X.Z.); m190310859@st.shou.edu.cn (H.C.); hanzheng@saas.sh.cn (Z.H.)

[2] Shanghai Key Laboratory of Protected Horticultural Technology, Laboratory of Quality and Safety Risk Assessment for Agro-products (Shanghai), Institute for Agro-food Standards and Testing Technology, Ministry of Agriculture, Shanghai Academy of Agricultural Sciences, Shanghai 201403, China; huangqingwen@zju.edu.cn

* Correspondence: niedongxia@saas.sh.cn (D.N.); whwu@shou.edu.cn (W.W.); Tel.: +021-37196975 (D.N.); +021-61900388 (W.W.)

† These authors contributed equally to this work.

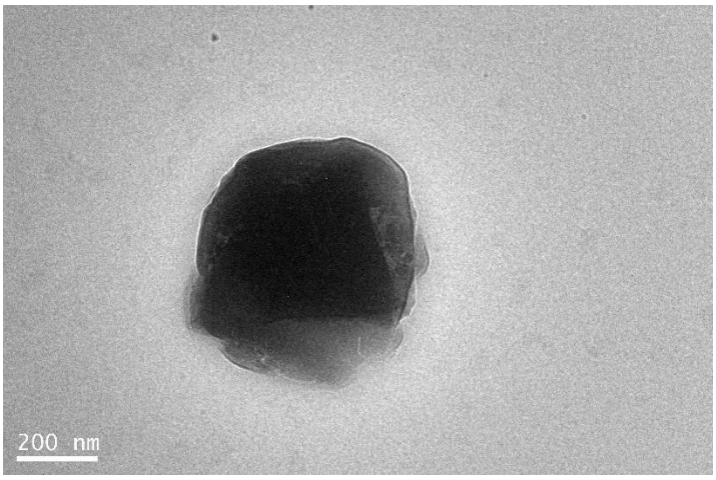

Figure S1. Transmission electron microscopy (TEM) of AP1/N-Cu-MOF

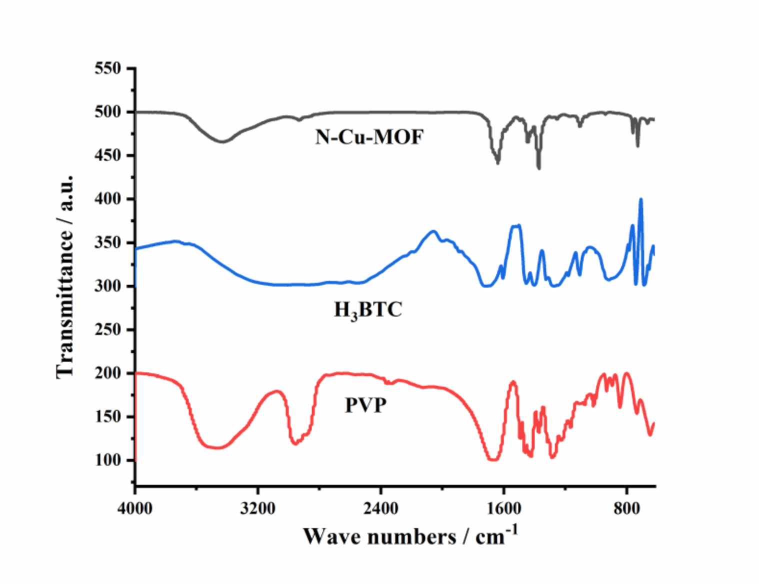

Figure S2. Fourier transform infrared spectroscopy (FT-IR) spectra of N-Cu-MOF, 1,3,5-benzenetricarboxylic acid (H_3BTC) and polyvinylpyrrolidone (PVP).

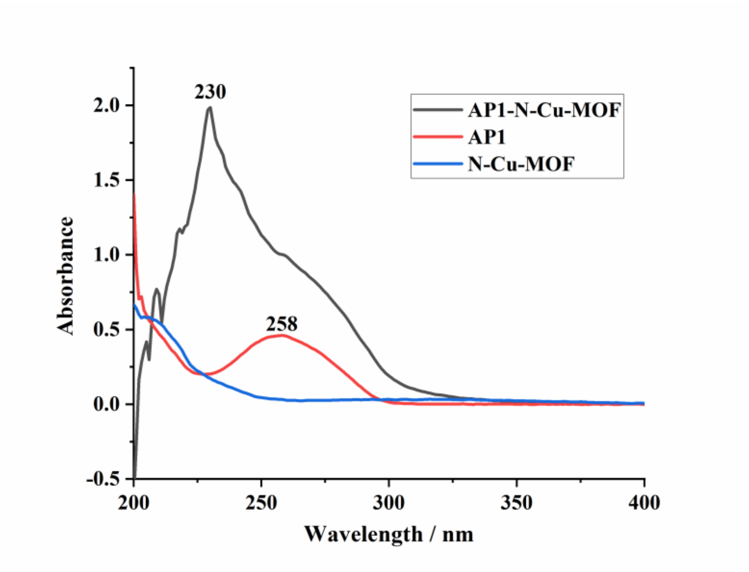

Figure S3. The UV-vis spectra of the N-Cu-MOF, AP1 and AP1-N-Cu-MOF.

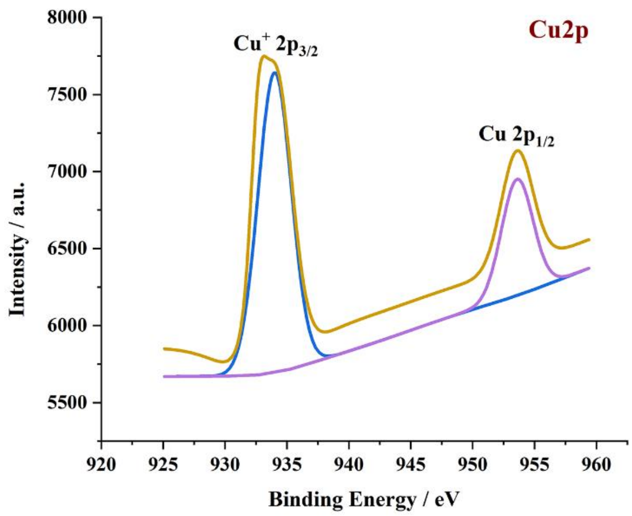

Figure S4. X-ray photoelectron spectroscopy (XPS) of AP1-N-Cu-MOF

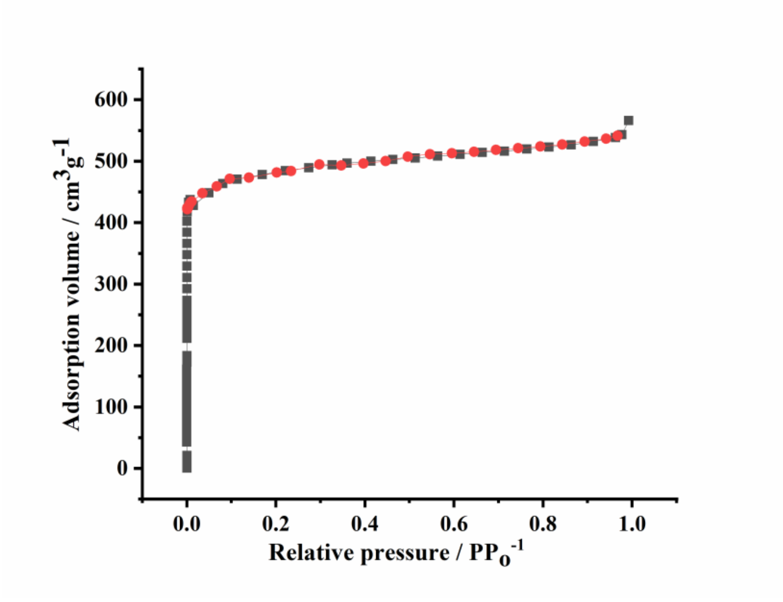

Figure S5. N_2 adsorption/desorption isotherms of N-Cu-MOF.

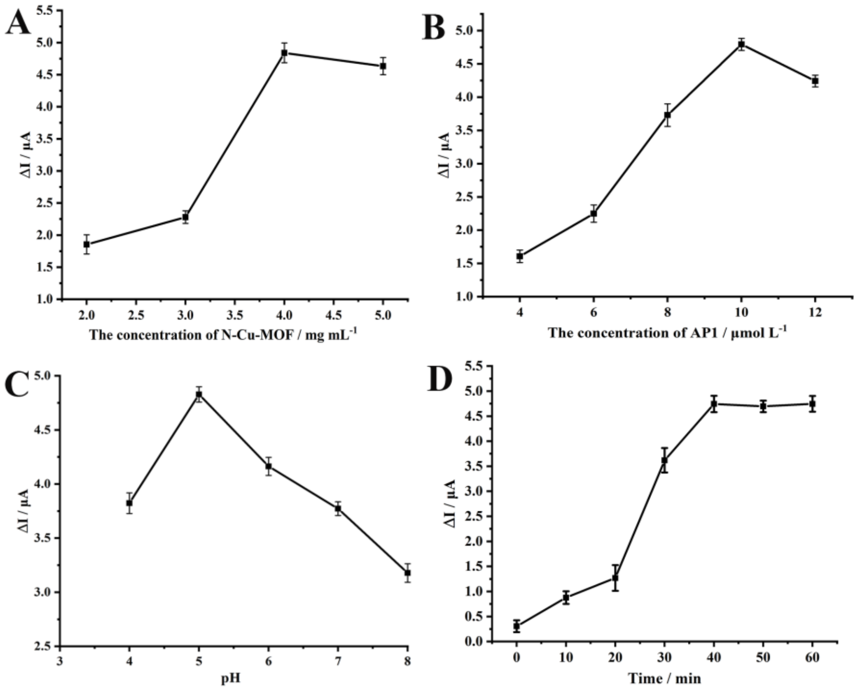

Figure S6. Optimization of AP1/N-Cu-MOF/GCE-based aptasensor. DPV responses with different concentrations of N-Cu-MOF (A) and AP1 (B), different pH values (C) and different incubation times (D) of AP1/N-Cu-MOF /GCE electrode in 1 mmol L^{-1} PBS containing 2 ng mL^{-1} DON.
